# Development and External Validation of a Deep Learning Algorithm to Identify and Localize Subarachnoid Hemorrhage on CT Scans

**DOI:** 10.1212/WNL.0000000000201710

**Published:** 2023-03-21

**Authors:** Antonios Thanellas, Heikki Peura, Mikko Lavinto, Tomi Ruokola, Moira Vieli, Victor E. Staartjes, Sebastian Winklhofer, Carlo Serra, Luca Regli, Miikka Korja

**Affiliations:** From the Department of Information Management (A.T.), Helsinki University Hospital, Helsinki, Finland; Department of Neurosurgery, University of Helsinki and Helsinki University Hospital (H.P., M.K.), Helsinki, Finland; CGI (M.L., T.R.), Helsinki, Finland; Machine Intelligence in Clinical Neuroscience (MICN) Laboratory, Department of Neurosurgery (M.V., V.E.S., S.W., L.R.), Clinical Neuroscience Center, University Hospital Zurich, University of Zurich, Zurich, Switzerland; Department of Neuroradiology (C.S.), Clinical Neuroscience Center, University Hospital Zurich, University of Zurich, Zurich, Switzerland.

## Abstract

**Background and Objectives:**

In medical imaging, a limited number of trained deep learning algorithms have been externally validated and released publicly. We hypothesized that a deep learning algorithm can be trained to identify and localize subarachnoid hemorrhage (SAH) on head computed tomography (CT) scans and that the trained model performs satisfactorily when tested using external and real-world data.

**Methods:**

We used noncontrast head CT images of patients admitted to Helsinki University Hospital between 2012 and 2017. We manually segmented (i.e., delineated) SAH on 90 head CT scans and used the segmented CT scans together with 22 negative (no SAH) control CT scans in training an open-source convolutional neural network (U-Net) to identify and localize SAH. We then tested the performance of the trained algorithm by using external data sets (137 SAH and 1,242 control cases) collected in 2 foreign countries and also by creating a data set of consecutive emergency head CT scans (8 SAH and 511 control cases) performed during on-call hours in 5 different domestic hospitals in September 2021. We assessed the algorithm's capability to identify SAH by calculating patient- and slice-level performance metrics, such as sensitivity and specificity.

**Results:**

In the external validation set of 1,379 cases, the algorithm identified 136 of 137 SAH cases correctly (sensitivity 99.3% and specificity 63.2%). Of the 49,064 axial head CT slices, the algorithm identified and localized SAH in 1845 of 2,110 slices with SAH (sensitivity 87.4% and specificity 95.3%). Of 519 consecutive emergency head CT scans imaged in September 2021, the algorithm identified all 8 SAH cases correctly (sensitivity 100.0% and specificity 75.3%). The slice-level (27,167 axial slices in total) sensitivity and specificity were 87.3% and 98.8%, respectively, as the algorithm identified and localized SAH in 58 of 77 slices with SAH. The performance of the algorithm can be tested on through a web service.

**Discussion:**

We show that the shared algorithm identifies SAH cases with a high sensitivity and that the slice-level specificity is high. In addition to openly sharing a high-performing deep learning algorithm, our work presents infrequently used approaches in designing, training, testing, and reporting deep learning algorithms developed for medical imaging diagnostics.

**Classification of Evidence:**

This study provides Class III evidence that a deep learning algorithm correctly identifies the presence of subarachnoid hemorrhage on CT scan.

The use of head CT imaging has continued to increase among adults during the 21st century.^1^ Moreover, in keeping with the increasing trend in favoring health care system integrations and consolidations, many countries have centralized radiology services during on-call hours. This leads to significantly higher volumes and complexity of on-call imaging cases, which in turn place increasing pressure on on-call radiologists. In fact, the overall on-call workload for radiologists has quadrupled in the past 15 years.^2^

Head CT scans are among the most frequently requested after-hour imaging studies in hospitals. Head CT scans outside normal working hours are mostly requested by emergency departments, where findings in an urgent head CT scan can change the patient's medical care. Perhaps the 2 most common patient groups who are imaged with an urgent head CT scan are patients with headache and stroke, for whom any delays in ruling out issues, like intracranial bleedings, may be tragic. Of the types of intracranial bleedings, undiagnosed subarachnoid hemorrhage (SAH) is among the most alarming ones because if the frequent cause, that is, a ruptured intracranial aneurysm, is left untreated, at least 75% of today's SAH patients die within a year.^3^ In middle-aged people, SAH deaths surpass the number of ischemic stroke deaths, and SAH deaths are in fact the most common type of stroke deaths in particularly middle-aged women.^4^

Although the rate of missed or misdiagnosed head CT findings is low, especially at academic centers, misinterpretations do happen, particularly during after hours, which are often covered by somewhat less experienced clinicians. It has been found that after-hour head CT reports provided by radiology residents at an academic large center were inaccurate in 4.6% of the cases.^5^ Fortunately, however, only 0.62% of the cases that were not identified or were inaccurately reported were intracranial hemorrhages (one-third of these were SAHs).^5^ These facts considered, the primary research question being addressed in this study was as follows: can a deep learning algorithm correctly identify and localize the presence of SAH on head CT scans.

## Methods

### Head CT Images for Deep Learning Training

We extracted noncontrast head CT images from the Helsinki University Hospital (HUH) Picture Archiving and Communication Systems (PACS) archive. First, using the HUH electronic medical records, we identified (based on the *ICD-10* category code I60) patients with SAH treated at HUH between 2012 and 2017 (Table 1). Similarly, we created a negative control group (no SAH on a head CT scan) by searching for patients who were admitted to the HUH emergency departments between 2011 and 2018 (Table 1), imaged with a head CT scan, and discharged home on the same admission day with a discharge diagnosis of headache (*ICD-10* codes R51 and G44.2). Because the head CT studies were performed with various multislice CT scanners, reconstructed slice thicknesses varied between 2 and 5 mm (Table 1). Similarly, the used imaging protocols varied by year, scanner, and hospital. Second, of the tens of thousands of potential cases and controls, we extracted noncontrast head CT studies of the identified patients and control subjects from the PACS archive, which contains more than 21 million digitally stored Digital Imaging and Communications in Medicine (DICOM) imaging studies. The extracted DICOM image series of patients with SAH consisted of axially reconstructed multiplanar reformatted (MPR) volumes imaged with 4 different CT scanners at HUH hospitals (Table 1). A similar image data set of control subjects originated from 5 different CT scanners (Table 1). In 2021, the HUH had altogether 19 different CT scanners. Third, after a slice-wise review of the extracted DICOM image series, 2 study authors (A.T. and M.K.) selected 98 MPR volumes corresponding to 96 patients with SAH, with 1 patient having 2 follow-up CT scans, and 985 MPR volumes corresponding to 949 control people with headache (no SAH detected on head CT scans), as 18 people were imaged at least twice. Apart from SAH, no other inclusion criteria were applied (such as demographics, findings of medical interventions [e.g., aneurysm clips, aneurysm coils, and ventricular catheters], image artifacts, image quality, image reconstruction methods, or image resolution) for the selected MPR volumes of patients with SAH.

**Table 1:**
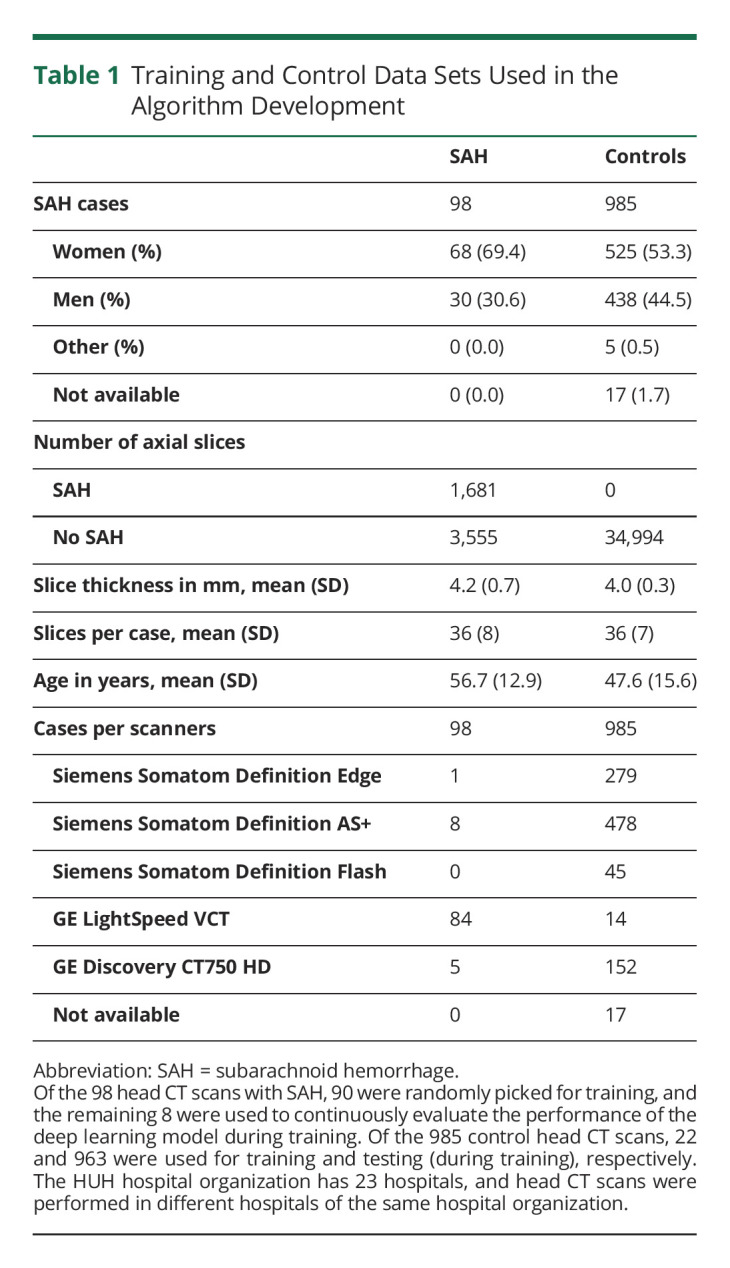
Training and Control Data Sets Used in the Algorithm Development

### Segmentation of SAH on Head CT Images

**Figure 1:**
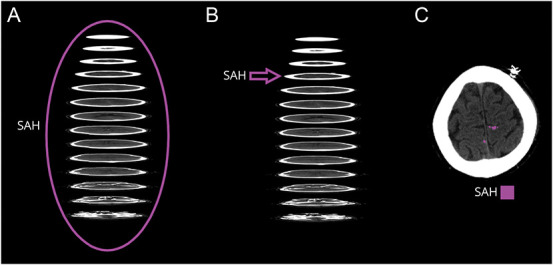
Basic Concepts of Image Annotations and Segmentations Illustrated In the patient-level annotation, the whole head CT scan (MPR volume) is classified as either positive or negative for SAH (A). In slice-level annotations, each of the approximately 30–40 axial slices of the head CT scan (MPR volume) is classified as either positive or negative (B). In a pixel-level segmentation, the aim is to delineate every positive pixel in every single slice (C). Segmentation of SAH is a time-consuming and laborious procedure, and therefore, medical images are mostly annotated (not segmented). MPR = multiplanar reformatted; SAH = subarachnoid hemorrhage.

Figure 1 and our previous publication^6^ present the concepts of annotation and segmentation. In brief, using the open-source utility dcm2niix, we converted the selected DICOM images to the Neuroimaging Informatics Technology Initiative (NIfTI) open file format for further processing. A trained medical image analyst (A.T.) performed a manual segmentation task (i.e., delineated SAH evident on head CT scans) using the open-source software ITK-SNAP ^e1^ and 3D Slicer.^e2^ Following this, the study neurosurgeon (M.K.) reviewed and adjusted the segmentations for the training set, but not for a data set segmented to assess a pixel-level algorithm performance. We performed adjustments to the segmented data only when a mutual (A.T. and M.K.) agreement was achieved. These segmentations (i.e., ground truths) were drawn only onto the axial MPR planes, since the axial MPRs are commonly used in clinical diagnostics.

### Preprocessing of Training Images

We downsampled the 512 × 512 image resolution to 256 × 256, which in other words downscaled the NiFTI image slices by a factor of 2 both in horizontal and vertical directions. In downsampling, we kept the original slice numbers of every scan. We clipped the intensities of the head CT scans using the window range of [0, 150] Hounsfield units. Following this, we divided the segmented and preprocessed NiFTI MPR volumes into training and test sets.

### Training of the Deep Learning Algorithm

In training, we used an open-source and standard 2-dimensional 5-level U-Net-type architecture,^7,8^ in which each level consisted of 2 convolutional layers followed by max-pooling on the downscaling path and upsampling on the upscaling sides. The number of feature maps per each level was 30, 60, 120, 240, and 480. Simplified, U-Net is a convolutional neural network that has been designed particularly for medical image segmentation. The U-Net architecture is based on fully convolutional layers, and therefore, it may be trained with fewer images yet yielding accurate segmentations. In training, the network learns to classify pixels as either positive or negative, based on segmented (i.e., every pixel including the lesion of interest outlined positive) training images. When the fed input image travels through each convolutional layer, the so-called feature maps are generated by superimposing different filters (i.e., mathematical functions) on the input image, and the output value of the filter function is called a feature map. The feature map size changes at each convolutional layer, and the network learns to identify lesion-specific image features. Following training, the network is fed with raw images (no segmentations), and the trained U-Net creates a segmentation mask (i.e., outlines identified lesions) as a visual output.

We used the training set simply in training of the algorithm to segment SAH, whereas the small test data set (8 head CT scans) was reserved for testing the trained model along the training process. Of the 98 head CT scans with SAH, we picked randomly 90 for training. Of these 90 MPR volumes with SAH, 23 were head CT scans taken on admission before any invasive treatments. The remaining 67 MPR volumes were postoperative, of which 40 included aneurysm clips and clip-related artifacts, 22 included aneurysm coils and coil-related artifacts, and 5 were volumes showing ventricular catheters. We used the remaining 8 of 98 MPR volumes of patients with SAH as the small test data set during training to continuously evaluate the performance of the model. Of the negative (no SAH) control group of 985 head CT scans, we used 22 for training.

### External Validation

For the external validation, we used 2 different data sets, namely Zurich and CQ500 data sets (Table 2). These data sets were not used in any training or testing phases. We assessed the algorithm's capability to identify SAH and reported the results based on patient- and slice-level annotations. We calculated the patient- and slice-level^9^ performance metrics of the Zurich data set. Because the CQ500 data set included only case-level (not slice-level) annotations, we calculated only patient-level metrics for the CQ500 data set.

**Table 2:**
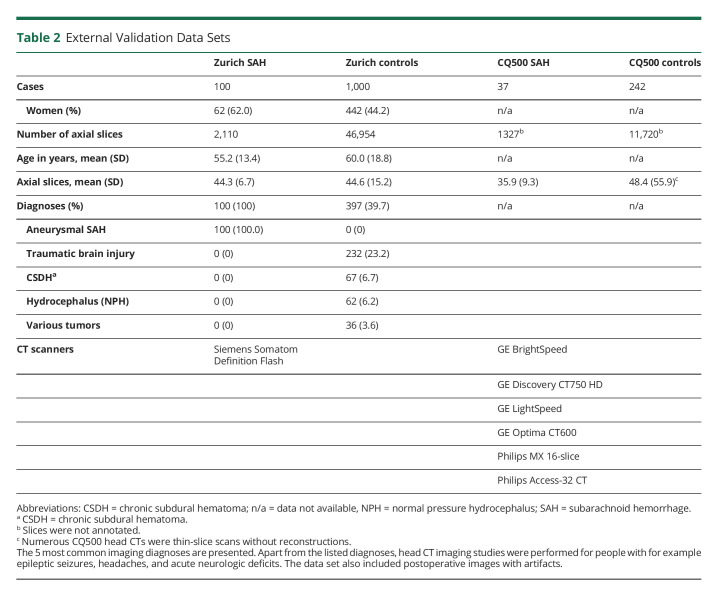
External Validation Data Sets

#### Zurich External Data Set

The coauthors from Zurich, Switzerland, selected and extracted head CT images of 100 consecutive patients with SAH and 1,000 consecutive control subjects (without SAH) from the PACS system of the University Hospital Zurich. To retrieve authentic real-world clinical data from another large hospital, we provided no other advice for the case and control selection process. Furthermore, we suggested no limitations apply for the CT scanners, imaging parameters, or imaging dates. We provided the coauthors with the trained algorithm, and the whole external validation process was conducted independently in Zurich. DICOM files were converted to the NiFTI files using the dcm2niix software. Preprocessing was performed with the scripts provided by the HUH research team, and these operations were run on an offline machine (Windows 10, AMD 1950X 32-Thread, 64 GB RAM, GTX 980 Ti). The algorithm's segmentations were visually checked using ITK-SNAP, and 2 raters (M.V. and V.S.) calculated the slice-level and patient-level performance metrics.

#### Open-Source External Data Set CQ500

A subset of open-source data set CQ500^9^ and its patient-level annotations from 3 raters as a ground truth was used as another external data set for validation. We rated the head CTs of patients as SAH cases when all 3 raters had annotated accordingly. Similarly, the head CT scan was considered negative (a control) if none of 3 rates found an intracranial bleeding in the scan. The final set consisted of 37 head CT scans with SAH and 242 head CT scans with no intracranial bleedings.

### Simulated Real-World Validation

Because the external validation set from Zurich originated from a large neurosurgery unit of a tertiary university hospital, which provides emergency care mostly for unconscious patients and patients already diagnosed with emergence lesions on head CT scans, we collected all consecutive emergency head CT scans imaged in September 2021 in 5 HUH hospitals, which have no neurosurgical services. These 5 hospitals and their case mix may therefore better resemble smaller on-call hospitals with head CT imaging facilities but no neurosurgical services. All collected CT scans were anonymized (no radiologic reports available), and annotated (slice-level) followingly for SAH by 3 coauthors (M.K., H.P., and A.T.). Similar to the CQ500 data set, an agreement of all 3 raters was considered a ground truth. After annotation, we analyzed all head CT scans using the algorithm.

### Pixel-Level Accuracy

Because neither the external validation data sets nor the real-world validation data sets were segmented, that is, they did not include pixel-level information about the true positives and negatives, 1 coauthor (A.T.) segmented additional 49 SAH cases as described earlier to test the model's pixel-level performance. The coauthor (A.T.) randomly selected 46 SAH of 1,237 noncontrast head CT studies of the identified patients with SAH (eTable 1, links.lww.com/WNL/C554) and included additional 3 SAH cases in which the diagnosis of SAH was originally missed, despite of positive head CT imaging findings.^10^ After segmentation, we analyzed all head CT scans using the algorithm.

### Postprocessing of Segmentations

As a sensitivity analysis, we applied simple postprocessing steps to the patient-level segmentations to reduce the number of false-positive cases. For the Zurich data set, we visually thresholded the number of cases where only 1 slice with a single pixel cluster was segmented positive. This single cluster in only 1 positive slice was considered negative (no SAH detected). For the CQ500 and HUH September 2021 data sets, we computed a Python script to evaluate the thresholding similarly, that is, if the case had only 1 slice with 1 segmented SAH cluster, the case was considered negative.

### Statistical Analyses

Patient-level metrics for the CQ500 and patient-, slice-, and pixel-level metrics for the HUH data sets were calculated automatically using Python scripts computed for these tasks. These metrics include sensitivity, specificity, false-positive rate, false-negative rate, and accuracy. We performed all statistical analyses with the Python package numpy and generated statistical plots with matplotlib.

### Ethical Considerations

The local institutional review board of HUH approved the retrospective data collection and study design and granted a waiver for acquiring an informed consent (HUS/365/2017, HUS/163/2019, and HUS/190/2021). According to Finnish legislation, no separate ethics committee approval is needed for retrospective studies that involve a secondary use of registry or archive data. We gathered all imaging data for algorithm training from the HUH, which consists of 23 separate hospitals and has a catchment area of approximately 2.2 million inhabitants. All 5 Finnish university hospitals, including the HUH, are publicly funded nonprofit organizations that provide tertiary health care services for all people living in Finland, regardless of socioeconomic status, insurance status, or race/ethnicity. Therefore, we believe that the HUH imaging data for algorithm training are not inherently biased or deliberately discriminative. We conducted the study in line with the Declaration of Helsinki.^11^ In Switzerland, the study was approved by the Zurich Cantonal Ethics Board (KEK Nr. 2020–02725) and the Data Governance Board of the University Hospital Zurich (Nr. DUP-66).

### Data Availability

Finnish health care data for secondary use can be obtained through FINDATA (Social and Health Data Permit Authority according to the Secondary Data Act). The used Finnish and Swiss health care data cannot be shared openly. Access to the CQ500 image set can be obtained through a website.^e3^ To share the algorithm code with others, we uploaded the code to the GitHub repository.^e4^ For the sake of reliability and transparency, we launched a website,^e5^ where anyone can test the algorithm performance by uploading head CT scans for analysis.

## Results

### External Validation

**Figure 2:**
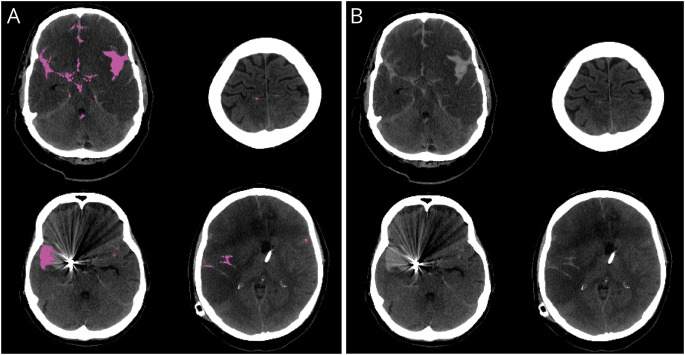
Examples of Segmentation Results (A) with the trained deep learning algorithm. The overall sensitivity of the algorithm was considered satisfactory, and it identified and localized SAH on axial head CT slices with extensive SAH (upper left image), sulcal SAH (upper right image), streaking clip artifacts (lower left image), and distortions (lower right image). The same images are presented in panel B without segmentations. SAH = subarachnoid hemorrhage.

The external validation data set consisted of 1,379 head CT scans (137 SAH cases) (Table 2). Few head CT scans from the external validation set were imaged with the same CT scanner (GE Discovery CT750 HD) that was used in imaging the training data set (Tables 1 and 2). The confusion matrices show the patient-level (Table 3) and slice-level (Table 4) results. Figure 2 shows 4 examples of how the algorithm identified and localized (i.e., segmented) SAH. The overall patient-level sensitivity and specificity were 0.99 and 0.63 for SAH, respectively (Table 3). The 1,379 head CT scans were composed of 49,064 reconstructed axial slices, of which 2,110 included SAH (Table 4). The slice-level sensitivity and specificity were 0.87 and 0.95, respectively (Table 4).

**Table 3:**
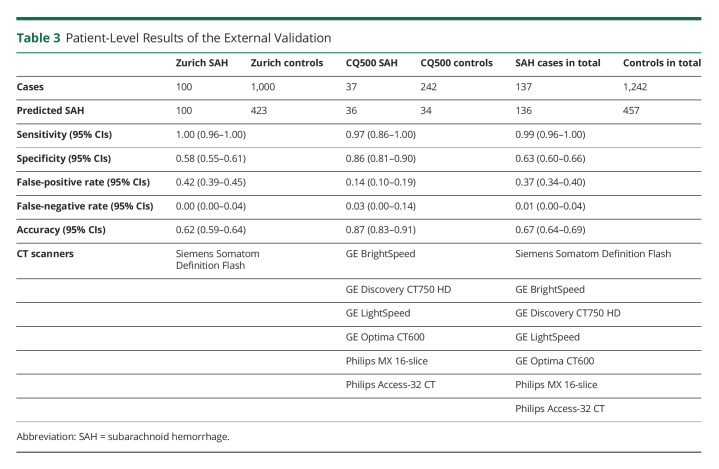
Patient-Level Results of the External Validation

**Table 4:**
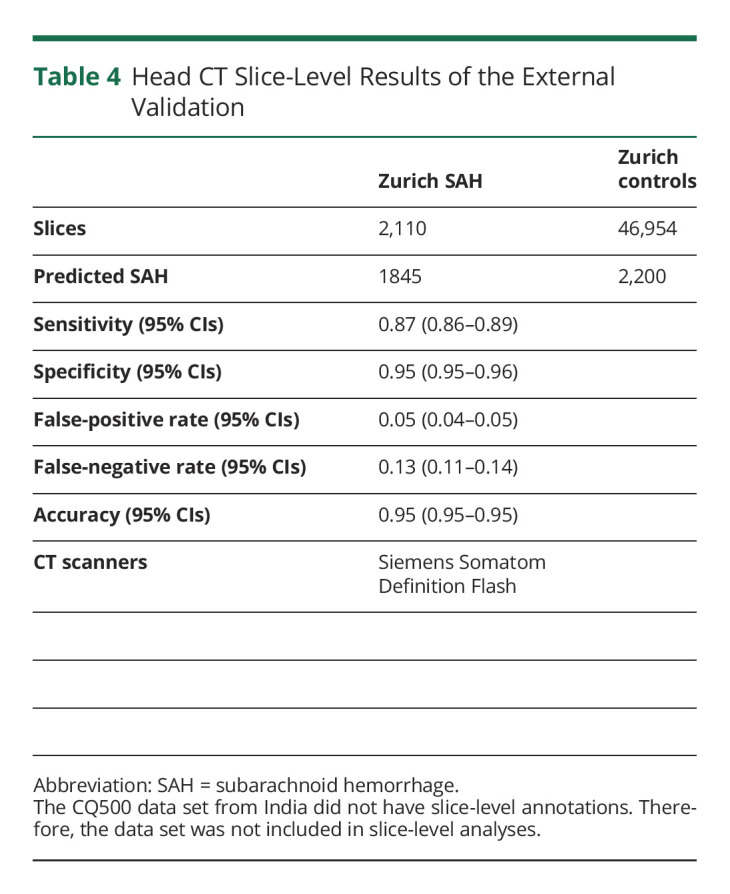
Head CT Slice-Level Results of the External Validation

The algorithm incorrectly classified 1 (0.7%) of 137 SAH cases as negative (Table 3, eFigure 1, links.lww.com/WNL/C554). At the slice level, the false-negative misclassification rate was 12.6% (Table 4). In terms of false positives, the results of the external validation showed a false-positive rate of 36.8% at the patient level (Table 3). Some of the false-positive cases were other abnormal findings than SAH. For example, of the 34 false-positive cases in the CQ500 data set, the algorithm falsely segmented 1 tumor, 1 artifact, 8 cases with calcifications, and 23 cases with no abnormal findings. Similarly, of the 423 false-positive cases in the Zurich data set, 138 (32.6%) were postoperative hematomas/hemostatic sealants, 54 (12.8%) ischemic lesions, 23 (5.4%) chronic subdural hematomas, and 21 (5.0%) tumors. At the slice level, the false-positive rate was 4.7% (Table 4).

### Simulated Real-World Validation

Of the 519 consecutive emergency head CT scans imaged during on-call hours in September 2021 in 5 smaller HUH hospitals without neurosurgical services, the algorithm identified all 8 SAH cases (Table 5). All CT scanners in the 5 smaller hospitals were newer and differed from those used in imaging the training data set. The patient-level sensitivity and specificity were 1.00 and 0.87, respectively (Table 5). The slice-level sensitivity and specificity were 0.75 and 0.99, respectively (Table 5). At the slice level, the false-positive rate was 1.2% (Table 5). Patient- and slice-level IRRs for 519 consecutive head CT scans were high (eTable 2, links.lww.com/WNL/C554).

**Table 5:**
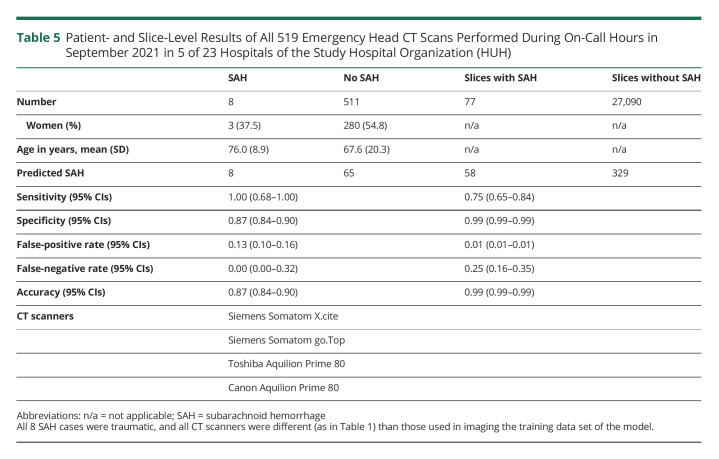
Patient- and Slice-Level Results of All 519 Emergency Head CT Scans Performed During On-Call Hours in September 2021 in 5 of 23 Hospitals of the Study Hospital Organization (HUH)

### Pixel-Level Accuracy

Because neither the external validation data set nor the simulated real-world validation data set included segmented images, we segmented and analyzed additional 49 SAH cases to test the model's pixel-level performance (eTable 1, links.lww.com/WNL/C554). The slice-level sensitivity and specificity were 0.78 and 0.97, respectively (eTable 3). At the slice level, the false-positive rate was 3.3% (eTable 3, links.lww.com/WNL/C554). The pixel-level sensitivity and specificity were 0.53 and >0.99, respectively (eTable 3, links.lww.com/WNL/C554). The pixel-level false-positive rate was <0.01%. Anecdotally, the algorithm also identified 3 SAH cases that were originally misdiagnosed in real life (eFigure 2, links.lww.com/WNL/C554). The CT scanners used in imaging the 49 SAH cases (eTable 1) were mostly the same as the scanners used in imaging the training set (Table 1).

### Online Validation Portal

We launched a website,^e5^ where anyone can test the accuracy of the SAH algorithm by uploading (drag and drop) noncontrast head CT scans for analysis. Axial MPR reconstructions should be converted (with any open-source DICOM-to-NiFTI converter) to the NiFTI format before uploading to fully anonymize the image data. The website is deployed, and the analysis of 1 head CT scan with 30–40 axial MPR slices takes around 30 seconds. The segmentation results are presented in color for visual inspection. The website is open for 180 days following online publication.

### Classification of Evidence

This study provides Class III evidence that a deep learning algorithm correctly identifies the presence of subarachnoid hemorrhage on CT scan.

## Discussion

The presented deep learning algorithm identified SAH correctly in 136 (99.3%) of 137 cases that were imaged with 7 different CT scanners in 2 countries (India and Switzerland). The only missed SAH was part of the CQ500 data set (eFigure 1, links.lww.com/WNL/C554). In terms of specificity, the algorithm incorrectly segmented SAH in 457 (36.8%) of 1,242 controls. The slice-level false-positive rate was 2,200 (4.7%) per 46,954 axial reconstructed head CT slices. A standard reconstructed head CT scan that is used in clinical diagnostics contains usually 30–40 axial MPR slices. If this algorithm was used in a clinical setting, the algorithm would falsely alarm clinicians about SAH in around every third normal (i.e., no SAH) head CT scan, and in these cases, 1–2 incorrectly segmented slices should be carefully inspected to revise the diagnosis. When designing algorithms for life-threatening emergency conditions, the sensitivity should optimally be close to 100% (i.e., no missed cases), although 100% sensitivity is a challenging goal even for human eyes. If such an algorithm also has a nonzero false-positive rate (less than 100% specificity), this obliges clinicians to inspect every positive case (also true positive cases). This may ensure that the algorithm is not replacing clinicians or radiologists but acts in real-life medical practice more like a collaborative colleague.

Trained imaging algorithms are frequently based on a high number of images. This same applies to algorithms for intracranial hemorrhages, which are often trained with a high number of annotated images.^12^ Our approach of using a small number of real-word training images with pixel-level segmentations instead of slice-level annotations may encourage others to adopt a similar strategy in training deep learning algorithms. When training images are segmented, large image data sets are less often needed, and deep learning projects become possible also in smaller medical centers. In addition to high-quality training, a validation process is of paramount importance. Although the sensitivity and specificity of internally validated imaging algorithms for SAH can be very high, their performance metrics when tested with external clinical data are often compromised.^12^ Because prior studies reporting deep learning algorithms that localize and identify SAH on head CT scans are scarce, any comparison between our and previous studies is difficult. In a seminal study on which the CQ500 data set is based and made available for the public, the highest patient-level sensitivity and specificity for identifying (not localizing) SAH were 92% and 90%, respectively.^9^ Patient-level results of another deep learning solution, the results of which were validated using an external data set of a reasonable (>100 positive cases) size, showed sensitivity and specificity of 85% and 97%, respectively.^13^ In a large external validation study of the world's first and most widely used commercial deep learning solution (which can only interpret thin 0.5–1 mm axial CT images of modern [>64 slices] CT scanners) for identifying intracranial hemorrhages, the patient-level sensitivity for identifying SAH was 93%.^14^ Apparently, many previous algorithms have probably been optimized not only for sensitivity but also for specificity at the expense of sensitivity. To avoid a deep learning model surpassing clinicians, our approach was to reach a very high sensitivity and a lower specificity, in which case a clinician deep learning model collaboration may become more likely. Of interest, 56% of false positives in our Zurich data set were in fact other pathologic lesions, such as postoperative hematomas. Indeed, the accuracy and particularly the false-positive rate of the algorithm can vary depending on natural confounders (other blood-containing pathologic lesions) and intended use (e.g., not intended to be used in postoperative imaging).

One of the study's strengths may be that the training data set included preoperative and postoperative artifacts and distortions. The training data set was imaged using different CT scanners, thus perhaps improving the generalizability of the algorithm. Moreover, because the external validation was conducted by using international data sets, and because the simulated real-world validation data set consisted of all consecutive head CT scans imaged in September 2021 in 5 different hospitals with 5 recently purchased modern CT scanners (none of which were used in imaging any other head CT scans in this study), these results may be generalizable. In addition, benchmarking our results is feasible with the open-source CQ500 data set. It is generally recommended to use not only open-source deep learning tools but also open-source data sets when available. We used open-source tools for segmentations, file conversions, and algorithm development. Despite having no influence on the selection process of images in India and Switzerland, these data sets may still somehow represent optimal cases for our algorithm, and therefore the results can be an overestimate. Because reproducing results based on machine learning algorithms is practically impossible by other research groups, we also launched a website,^e5^ where anyone can test the performance of the algorithm by uploading head CT images in a NiFTI format (i.e., anonymized data) for validation. Moreover, many deep learning algorithms are incapable of illustrating, visualizing, and delineating abnormal imaging findings, whereas the presented algorithm highlights SAH. This visualization may ease and fasten the image interpretation.^15^ As a further matter, the used U-Net architecture is small and can therefore be deployed on computers and devices with little computing power. Finally, we shared the algorithm for research purposes and further development in GitHub.^e4^ Maybe even low-income countries can benefit from this solution.

The training data set consisted of people living in Finland. Because Finns are genetically considered an independent subpopulation of the European population,^16^ our algorithm may be biased. Particularly, the false-positive rate varied between data sets. Whether this depends on the race remains to be studied. In addition, we lack a Conformité Européenne (CE) mark for the algorithm, which belongs to high-risk classes (IIa, IIb, and III) of medical devices. Such accredited assessment and issuing the CE mark are expensive and time-consuming processes, and many university hospitals have little capability to productize medical devices. Moreover, because only 1 data set was segmented (i.e., every pixel with SAH was delineated), and this data set came from Finnish hospitals, we were able to calculate pixel-level performance metrics only for this data set (eTable 3, links.lww.com/WNL/C554). Because a ground truth segmentation for SAH on head CT scans is a rather impractical measure (i.e., it is challenging for experts to agree about true positives and negatives at the pixel level), pixel-level results are clinically less meaningful and seldomly, if ever, reported. However, the pixel-level results were satisfactory (eTable 3, links.lww.com/WNL/C554), and false-positive segmentations consisted of small clusters of incorrectly segmented pixels (results not shown). Inspecting small clusters of false-positive pixels (the pixel-level false-positive rate <0.01%) in a few slices (the slice-level false-positive rate 4.7%) per head CT volume (the patient-level false-positive rate 36.8%) puts unlikely a strain on radiologists or clinicians. However, depending on the intended use, the number of false-positive pixels could be decreased with simple postprocessing steps (e.g., by ignoring dispersed small pixel clusters) and further development. Finally, we did not test the algorithm prospectively in any emergency department setting. This is an unfortunate but most common shortcoming in developing medical imaging algorithms, as implementing a research algorithm in a hospital PACS system and clinical workflow is legally and technically a cumbersome process, which in addition to financial resources may require close collaboration with the PACS solution provider. However, the simulated real-world validation data set with all consecutive cases from 5 hospitals resembled a prospective study setup in this context. On the other hand, the patient-level balance between positive and negative findings varies significantly between every hospital and institution, and therefore, even our real-world sensitivity and specificity figures may be imperfectly generalizable.

In conclusion, a similarly trained simple SAH algorithm could serve as a useful tool to assist in the diagnosis of SAH in a clinical setting. Because the presented algorithm lacks the CE mark, the algorithm cannot yet be used for a clinical purpose.

## Glossary

CE: Conformité Européenne
DICOM: Digital Imaging and Communications in Medicine
HUH: Helsinki University Hospital
*ICD*: *International Classification of Diseases*
MPR: multiplanar reformatted
NIfTI: Neuroimaging Informatics Technology Initiative
PACS: Picture Archiving and Communication Systems
SAH: subarachnoid hemorrhage
U-Net: neural network
